# Technologies Used in Production Systems for Santa Inês Sheep: A Systematic Review

**DOI:** 10.3389/fvets.2022.896241

**Published:** 2022-05-31

**Authors:** Andréia Santana Bezerra, Marcos Antônio Souza dos Santos, José de Brito Lourenço-Júnior

**Affiliations:** ^1^Institute of Veterinary Medicine, Postgraduate Program in Animal Science (PPGCAN), Federal University of Para (UFPA), Federal University of the Amazon (UFRA), Brazilian Agricultural Research Corporation (EMBRAPA), Castanhal, Brazil; ^2^Socio-Environmental and Water Resources Institute (ISARH), Federal University of the Amazon (UFRA), Belém, Brazil

**Keywords:** Brazil, lamb, ewe, technological tools, small ruminants

## Abstract

This study identifies the number of publications that presented technologies used in the production systems of Santa Inês sheep in the last 5 years (2017–2021) carried out in Brazil. Therefore, the objective was to identify where we are in terms of knowledge about technologies in different fields (health, reproduction, animal breeding, behavior and welfare, nutrition and feeding, forage and pasture, carcass and meat quality, and economics and management of livestock systems). After rigorous selection, 114 studies were appointed and classified by knowledge field, and the main approaches within each theme were evaluated, pointing out research gaps. Most technologies have been in northeastern states. However, government agencies should develop public policies to disseminate techniques in rural areas because the production system in this region is still subsistence. This study highlighted the need for works that present management practices and tools that impact the improvement of animal welfare. Agro-industrial by-products have been widely used as an alternative for sheep feeding. However, economic feasibility analyses are recommended with these foodstuffs to substantiate their use as an option to reduce production costs. There is a lack of research allusive to the management of production systems, especially those related to estimates of economic feasibility indicators.

## Introduction

Animal production systems such as meat sheep involve several production factors. They can be classified into three groups such as land (represented by areas of native and cultivated pastures), labor (human resources), and capital (includes machinery, equipment, facilities, and technological innovations) (1). The agricultural technology concept refers to the knowledge, techniques, and artifacts that enable the use of technological elements in livestock and agricultural activities. It involves techniques to control the growth and harvesting of an animal and to improve animal production, such as new ways of managing production systems (process innovations), management practices, improved breeds of animals, and changes in agricultural practices (2–4).

These technologies are used in different knowledge fields such as genetic improvement, health, nutrition, and others. Thus, the low adoption of these technological innovations generates incipient productivity levels (5, 6), affecting the entire production chain.

The Brazilian sheep herd represents ~1% of the world and the Northeast region stands out with 66.7% of the national herd. Following soon after is the South region with 21.2%, and a small portion is represented by the Midwest (5.4%), North (3.5%), and Southeast (3.2%) (7). However, in most parts of Brazil (mainly in the North and Northeast regions), sheep farming is predominant in family agriculture (8–10). This type of farming uses low or non-technological innovations and has low productivity.

Among the different sheep breeds in Brazil, Santa Inês has often been used for meat production due to its size and growth rate compared to other hairless breeds (11). In addition, its good adaptability to the Brazilian tropical conditions has given it prominence, attracting the attention of farmers to its productive potential (12). Thus, highlighting technologies used in the farming systems with this breed becomes crucial for developing activities.

Thus, the objective of this study was to identify the state of the art in research on production systems for meat sheep of the Santa Inês breed and answer the following questions: Where are we in terms of knowledge about technologies in different fields? How many studies executed in Brazil and published in the last 5 years demonstrate techniques applied to the production system of Santa Inês sheep?

## Materials and Methods

This type of study is applied, according to the proposal of Silva and Menezes (13), and aims to promote knowledge that would be used practically and assist in decision-making. Furthermore, its objectives are classified as exploratory and descriptive so the researchers can evaluate the data in a holistic and interpretative way. And finally, the applied technical procedures frame it as a systematic literature review.

This study followed some steps proposed by Cronin et al. (14), which were: (I) formulate the research question; (II) identify the databases and define the search strategies; (III) select and access the literature; (IV) assess the quality of the literature included in the assessment; and, (V) analyze, synthesize, and disseminate the results.

### Formulate the Research Question (I)

**Research Question:** What is the scientific research scenario about technologies and innovations used in the production systems for the Santa Inês sheep breed in the last 5 years (2017 to 2021) in Brazil?

### Searched Databases and the Search Strategies (II)

The conduction of a systematic review depends on the scope and quality of the included studies (15). Thus, to recover scientific articles of proven quality, we sought to use only electronic bases that retrieve journals that perform peer review of a manuscript, which were national and international. The bases used were Scopus, Scielo, Web of Science, and Portal de Periódicos Capes. According to Levy and Ellis (16), this step is called entry.

Next, the descriptors and Booleans used as search guidance in each database were determined. The Portuguese words were translated into English, and the search proceeded. The search method varied a few times among databases because they have some peculiarities.

Boolean operators were for establishing selection criteria and thus retrieving the maximum number of papers related to the theme. The *AND* operator was inserted between two words for searching articles that contain both (e.g., *animal AND reproduction*). Whereas, the Boolean operator *OR* was used to extend the search beyond one word (e.g., *sheep OR ram* searched for articles containing one or the other). Keywords between quotation marks were used for refining the search. Therefore, articles were retrieved only if the words appeared together.

After the search using the keywords and Booleans, it was proceeded to refine the results obtained through database restriction tools. In all databases, the time frame ranges from 2017 to 2021.

#### Scopus E Web of Science

The search in the Scopus and Web of Science databases was similar. The following keywords and combinations were used through Booleans to refine the search and exclude useless articles.

The first search in the main section was common to all fields and the *article title* option was selected (searching for words contained only in the article title) with the following combination: *sheep OR sheep OR lamb OR ram OR ewe*.

In the second session, the search was refined in *article title, abstract, and keywords/topic* with the following combination according to the field: *animal AND health, animal AND reproduction*, “*animal breeding,” animal AND welfare, animal AND nutrition*, “*grazing system,” “meat quality” AND carcass*.

Due to the scarcity of papers about livestock systems management, the second session was broader in this field by selecting the *all field* option (searching for the term throughout the article). The following combination was used: “*production system” AND economic AND cost*.

In the third session, the *all field* option was selected and its objective was to retrieve experiments performed with the Santa Inês breed. For this, the following combination was entered: “*Santa Inês” OR “Santa Ines” OR “Santa Inez.”*

In the fourth section, the option *all field* was selected, and the word *Brazil* was inserted for all fields. The objective was to reinforce the search for studies conducted in Brazil.

After obtaining the results, only scientific articles were selected, excluding review articles, books, conference papers, chapter books, notes, erratum, letters, and editorials. The databases also offered the option to choose the place of the experiment, so we selected the option of studies conducted in Brazil, refining the search only to the studies in English and Portuguese.

#### Periódicos Capes

The Periódicos Capes database has a broader coverage as it encompasses the other cited. However, it also differs in part as to the search criteria. The base gives four refinement options and searches the word within these, which are: *any field* (any part of the article), *title* (is contained in the title), *author/creator* (among the authors), and *subject* (is part of the document subject).

The first, third, and fourth sessions were similar with the Scopus and Web of Science bases, with the same combination of words, marking the option *title, any field*, and *any field*, respectively. In the second session, the search was different from the other databases because of its broader scope, and then quotation marks were added. However, the option “any field” was selected to avoid restricting the search further. The combination made is as follows: “*animal health,” “animal reproduction,” “animal breeding,” “animal welfare,” “animal nutrition,” “grazing system,” “meat quality” AND carcass, “production system” AND economic AND cost*.

The database also gives the option to select year and language, but not an experiment place. Another option would be to choose only peer-reviewed journals, and in the present work, this option was selected.

#### Scielo

This database differs in the selection classification options and subdivides into the following topics: *all indexes, publication year, author, funder, journal, abstract*, and *title*.

The first and third sessions did not differ from the other databases. In the second session, the *all indexes* option was selected, using the same combination of Scielo and Web of Science.

There was no fourth section, aiming to make the search more wide-ranging because of the papers' retrieval reduction on this base. We also selected only scientific articles, English and Portuguese languages, and the place of study (Brazil).

### Inclusion and Exclusion Criteria (III)

The articles retrieved from databases were selected according to the criteria described below. The criteria established were based on the objective proposed by the research. We selected only studies conducted in Brazil and excluded those that did not describe the place where the experiment was conducted.

The focus should be on meat sheep, using only the Santa Inês breed. Studies that involved crossbreeding, combined other breeds or species, or used no defined breeds were excluded. The only study selected that used a species other than sheep was the pasture system because other species such as cattle characterized a technology for endoparasite control.

They should obligatorily present a technique and/or innovation related to the different production systems for meat sheep. Clinical studies, case reports, or those that did not highlight a technology were also excluded.

After being selected according to the mentioned criteria, the articles were grouped into eight different knowledge fields (health, reproduction, animal breeding, behavior and welfare, nutrition and feeding, forage and pasture, carcass and meat quality, and economics and management of livestock systems), according to Table 1.

**Table 1:** Classification of studies according to the scopus.

**Scopus**	**Studies' classification method**
Animal health	They evaluated some sanitary handling techniques or used technology on animals' health. The main work focus should be on health.
Animal reproduction	They were related to the use of technologies proposed to affect the reproductive efficiency of both males and females.
Animal breeding	They involved techniques that aimed to assist in the selection of superior animals.
Animal behavior and welfare	They demonstrated tools to contribute to the animals' well-being and the study of their behavior, taking into consideration the five freedoms (freedom from hunger and thirst; freedom from discomfort, pain, disease, and injury; freedom to express the natural behaviors of the species; and freedom from fear and stress).
Animal nutrition and feeding	They used some techniques for animal performance evaluation, such as weight, weight gain, consumption, digestibility, and/or feed efficiency.
Forage and pasture	They used some pasture management techniques or different grass or legume species.
Carcass and meat quality	They used technologies from different fields focused on the meat and/or carcass quality assessment.
Economics and management of livestock systems	They evaluated the production cost and profitability of the system and economic variables.

*Source: research data*.

### Critical Analysis of the Selected Studies (IV)

The articles chosen in the previous step were read in full to extract relevant aspects of the objectives, methodology, results, and conclusions. This analysis was also related to the quality of the studies, which should have a detailed description of the methodology (without experimental problems) and conclusive results with a thorough discussion.

### Summary of the Results (V)

The presentation of the results focused on describing the main characteristics of the studies, highlighting the places where they were conducted, the technologies, and the approaches used in the systems.

## Results

**Figure 1:**
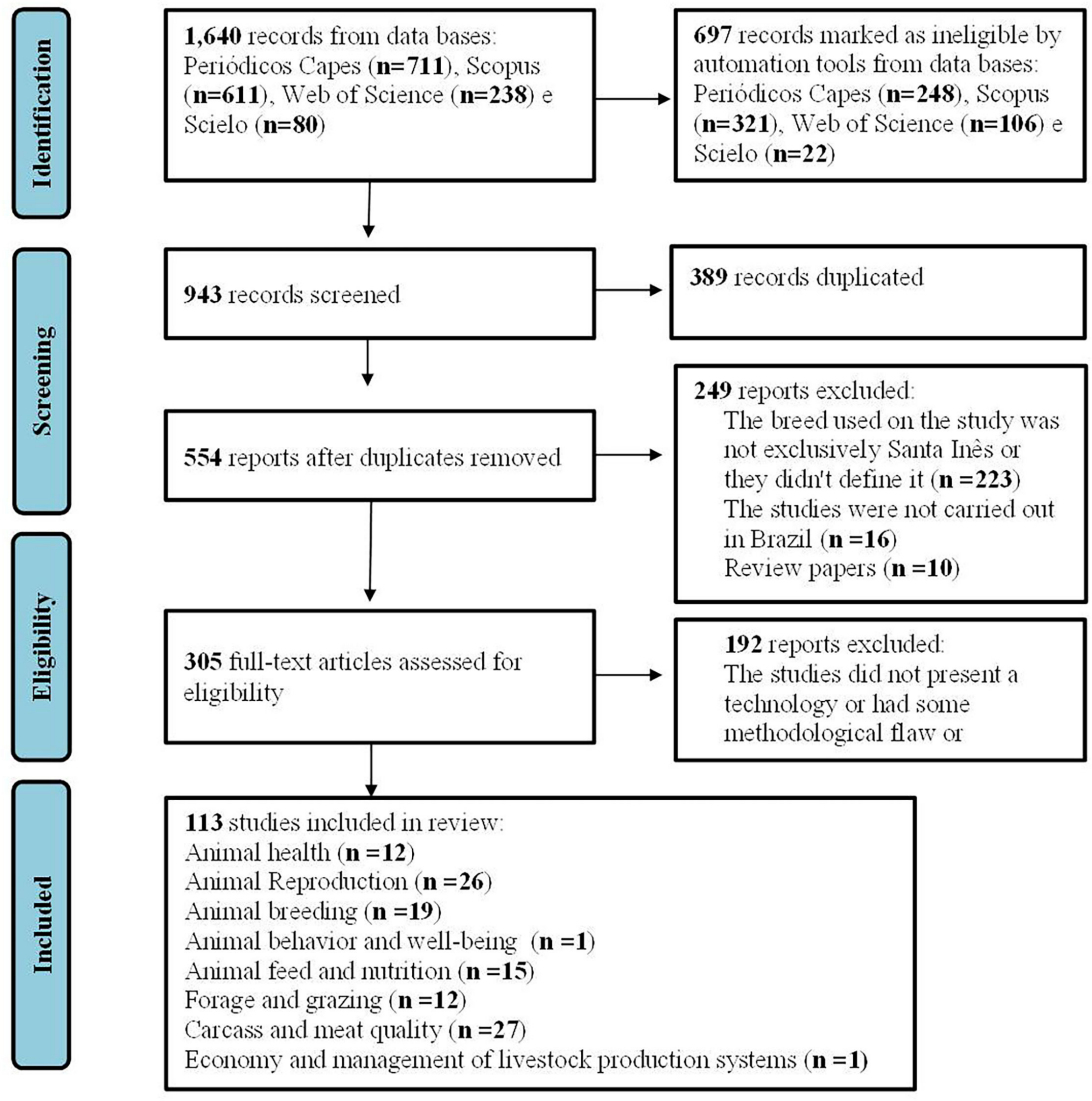
PRISMA flow diagram (animal study). Summary of the literature search, screening, and selection of potential studies. The PRISMA flow diagram represents the literature search in three different electronic databases, which are Capes Periodicals, Scopus, Web of Science, and Scielo, followed by screening and inclusion of eligible studies for systematic review. Source: Research data.

The PRISMA flow diagram (17) for the literature search summary, screening, and selection of potential studies is shown in Figure 1.

After the search using the keywords and Booleans, 1,640 papers were retrieved. Subsequently, when we used the exclusion criteria of the databases, the studies were reduced by 42.5%, totaling 943 scientific articles, from which 113 were selected (Figure 1).

**Figure 2:**
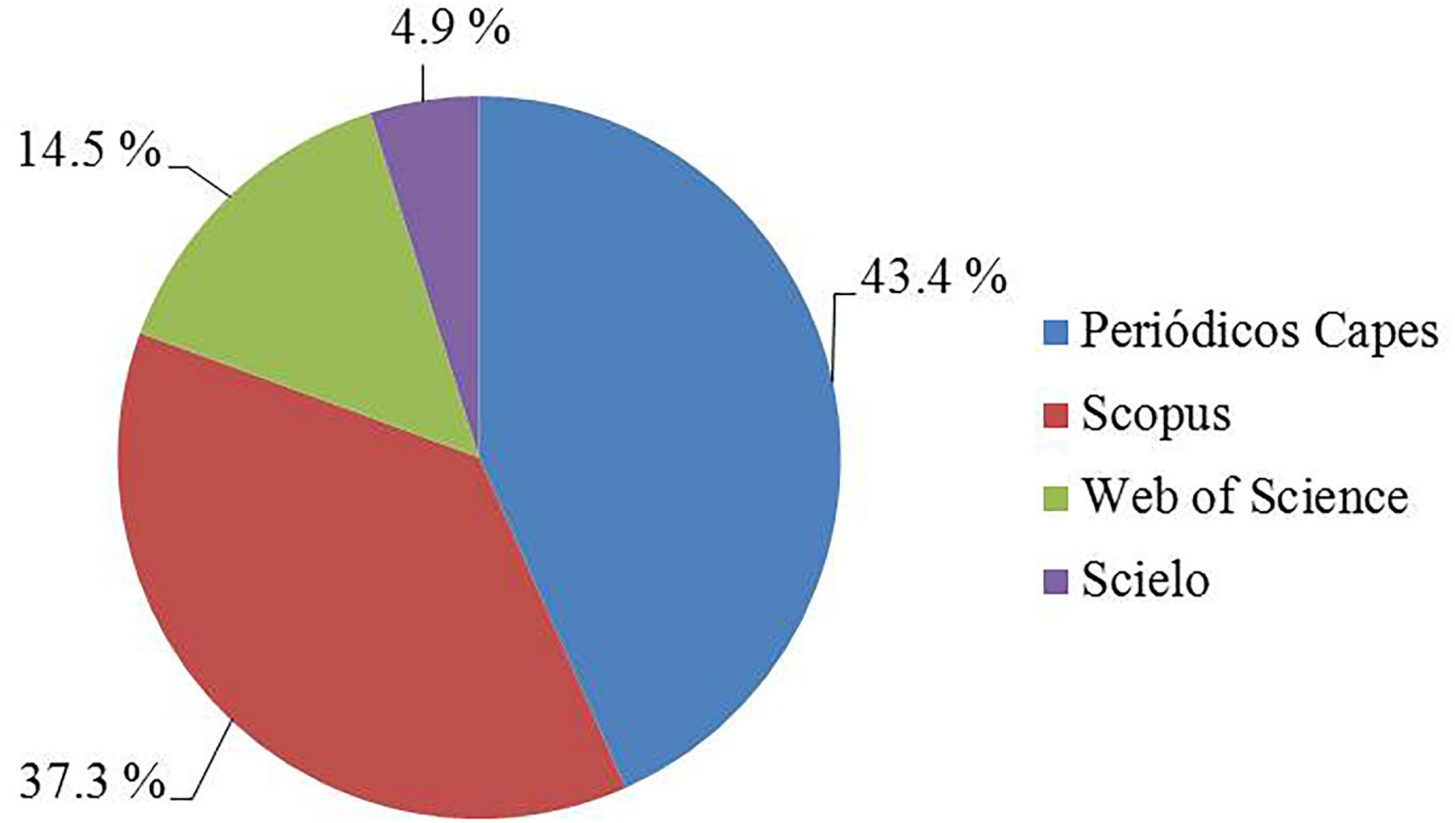
The proportion of articles retrieved from the databases from 2017 to 2021. Source: Research data.

Periódicos Capes and Scopus databases took up the most studies, totaling more than 80%. It occurred because these databases have more extensive databases. More discretely, the Web of Science database retrieved 14.5%. It is a reasonable quantity to be evaluated. On the other hand, few studies were retrieved from Scielo (2.2%) (Figure 2).

**Figure 3:**
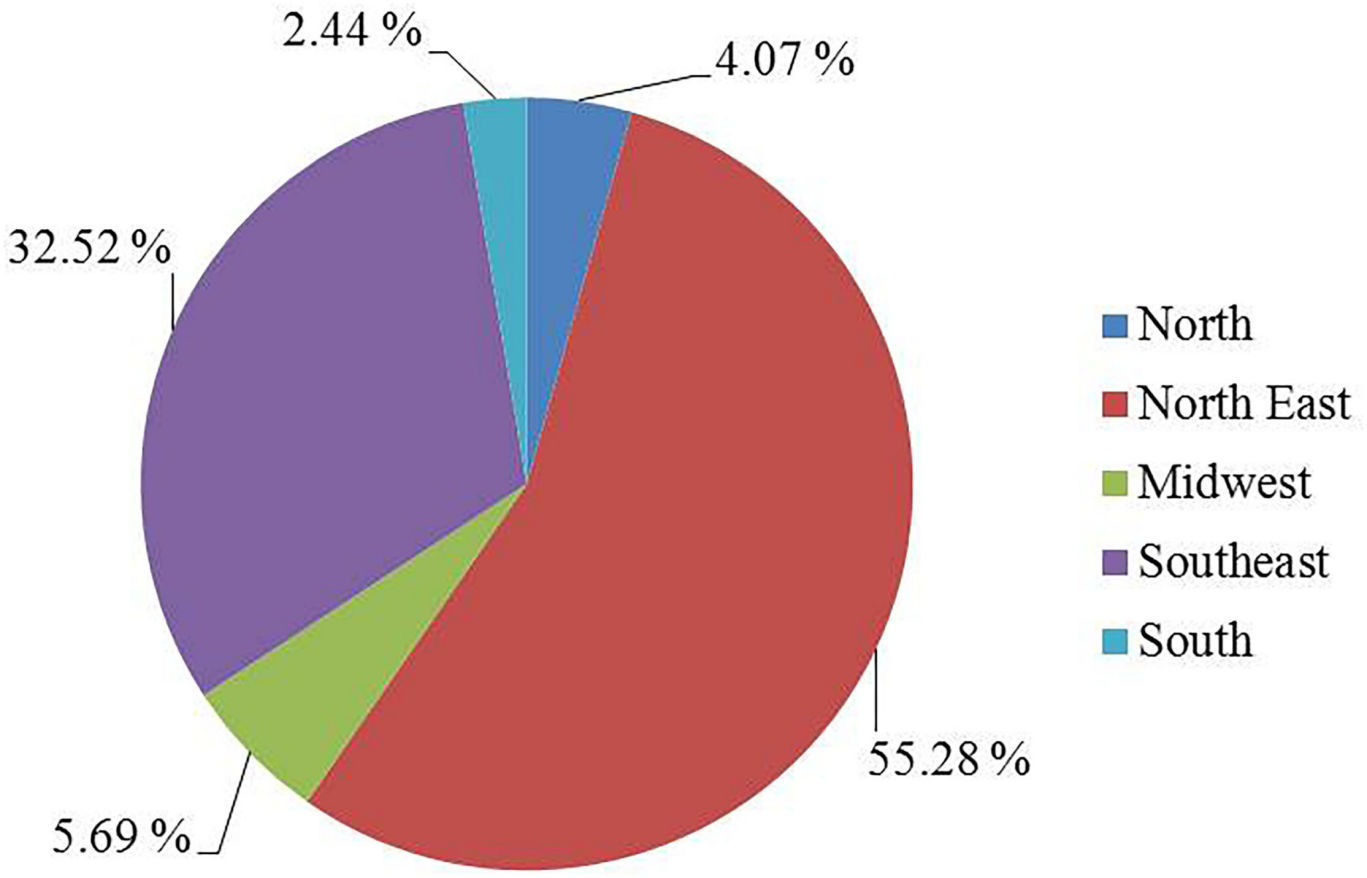
Geographical distribution of the studies. Source: Research data.

As for the location, most of the work was concentrated in the Northeast (55.28%), followed by the Southeast (32.52%). On the other hand, a reduced amount of research in recent years has been developed in the Midwest (5.69%), North (4.07%), and South (2.44%) regions (Figure 3).

Table 2 illustrates the categorization of all selected research. Most of the published papers from the last 5 years focused on carcass and meat quality (23.9%) and reproduction (23.0%). Soon after, the frequency concentrated on the so-called triple of the animal science with studies focused on animal breeding (16.8%), nutrition (13.3%), and health (10.6%), with a considerable number of selected works also in the field of forage (10.6%). Scarce studies were verified involving animal behavior and welfare (0.9%) and economics and management of livestock systems (0.9%).

**Table 2:** Studies' distribution according to the scopus.

**Scopus**	**%**	**Authors**
Animal health	10.6	(18–29)
Animal reproduction	23.0	(30–55)
Animal breeding	16.8	(56–74)
Animal behavior and welfare	0.9	(75)
Animal nutrition and feeding	13.3	(73, 74, 76–88)
Forage and pasture	10.6	(89–99)
Carcass and meat quality	23.9	(100–125)
Economics and management of livestock systems	0.9	(126)

*Source: research data*.

### Animal Health

Some technologies employed to promote a positive impact or not cause damage to the health of Santa Inês sheep are described in Table 3. The highlight was for dietary strategies such as propolis extract, probiotics (*Bacillus thuringiensis*), biopolymer (chitosan), and by-products. Also, the use of drugs such as an antibiotic and hormones could be evidenced. Other techniques focused on endoparasites' control and combat (Table 3).

**Table 3:** Technologies used with impacts on animal health of Santa Inês sheep, 2017–2021.

**Technology**	**Description**	**Primary effect or outcome**	**Authors**
Dietary strategy	Red propolis Extract (RPE)	Benefits to defense cells (3 g RPE/ ewe/ day)	(21)
	Probiotic (*Bacillus thuringiensis*) -BT	Does not cause intoxication (2.5 × 106 spores of BT/kg body weight-BW)	(22)
	Chitosan and cotton husk	Does not affect the metabolic and ruminal health (136 mg/kg BW)	(23)
	Cottonseed cake (CC)	No health effect 0, 140, 280, and 420 g of CC kg /BW;	(24)
	Guava (*Psidium guajava* L.) by-product	Supports metabolism (inclusion of up to 30% in the diet)	(25)
	Protein levels	Causes resistance to helminth (19% protein)	(29)
	*Acacia mearnsii* (AM)	Controls helminth infestation (15 g of AM/animal day)	(30)
Pharmaceuticals	Recombinant bovine somatotropin (rbST)	Reduces the risk of pregnancy toxemia (160 mg rbST)	(26)
	Antibiotic (gentamicin-GEN)	Does not prevent new mammary infections (250 mg of GEN)	(27)
Selective treatment tool	FAMACHA© system	Helps in the helminth infestation identification	(31, 32)
Biological control	Nematophagous fungi (*Arthrobotrys conoides, Arthrobotrys robusta, Duddingtonia flagrans*, and *Monacrosporium thaumasium*)	Reduction of endoparasite larvae in pasture (0.2 mg of fungus per kg BW)	(28)

*Source: research data*.

### Animal Reproduction

In the reproductive field, some studies addressed techniques used to increase reproductive efficiency in males and females, with a higher frequency of studies in females. Semen additives, dietary strategies, hormones, handling tools, ultrasound, and infrared thermography devices summarized the main techniques to support the increase in male or female sheep reproduction (Table 4).

**Table 4:** Technologies used to increase reproductive efficiency of Santa Inês sheep, 2017–2021.

**Technology**	**Description**	**Primary effect or outcome**	**Authors**
Dietary strategy	Palm kernel cake (PKC)	Does not affect seminal quality (up to 45%)	(37)
	Concentrate supplementation	Maintains the reproductive performance of females (1.5% of BW)	(39)
	Crude glycerin (GB)	Does not affect reproductive performance before and after the breeding season (10% of GB in dry matter-DM)	(42)
		Improves energy balance in pre and post parturition (10% GB in DM)	(43)
	ADE Vitamins	Improves energy metabolism in females	(40)
	Protected fat (PFAT)	Not recommended as it reduces the probability of gestation (5.5 and 13.5% PFAT per 60 days)	(41)
Semen additives	Dimethylacetamide (DMA) combined or not with glycerol (GLI)	Can be used for semen freezing without damage (max 5% DMA and max 6% GLI)	(34)
	Dimethylacetamide (DMA) combined with trehalose (TRE)	Can be used for semen freezing without causing major damage (3% DMA plus 100 mOsmol/L of TRE)	(35)
	Adenosine - ADA (a purinergic nucleoside)	Improves seminal parameters at 5°C (05 and 0.75% of ADA)	(36)
Sperm selection methods	Swim-up, Percoll, mini-Percoll, centrifugal sperm wash	Swim-up provides high values of sperm parameters and Percoll recovers more cells	(38)
Intravaginal devices	Re-use of progesterone devices	Can be used, without negatively affecting reproductive efficiency	(44)
	Human intravaginal tampon embedded with natural progesterone	Can be used without adversely affecting pregnancy rate	(45)
Hormones	Equine chorionic gonadotropin (eGC)	Effective in long duration protocols (200 to 400 IU eCG)	(46)
	Human chorionic gonadotropin (hCG)	Induces corpus luteum formation (250 IU eCG)	(47)
	Gonadotropin-releasing hormone (GnRH)	Efficient in synchronizing ovulation (10 to 25 μg of GnRH)	(48)
	Follicle-stimulating hormone (FSH)	Effective in ovulatory response and embryo production (100 or 200 mg of pFSH (FSH from swine) combined with a dose of eCG)	(49)
		Successive administration of FSH was not effective	(50)
	Oxytocin (OCI)	Effective in cervical dilatation without affecting the viability of the corpus luteum (100 IU of OCI)	(51)
	Estradiol benzoate (BE), d-cloprostenol (CL) and oxytocin (OCI)	Can be used, care must be taken with the timing of administrativo (37.5 μg CL, 50 IU OCI 1 mg BE)	(52)
	Dexamethasone	Effective for concentrating labor and facilitating handling (8 and 16 mg)	(127)
Management tools hours	Male effect combined with temporary weaning for 24	Improves reproductive performance of ewes	(54)
		Promotes preovulatory LH peaks without compromising pregnancy rate	(55)
Embryo transfer No difference	Embryo transfer or natural mating	Not significant difference	(53)
Ultrasonography devices	Manual or automatic Doppler	Dopper is more accurate	(128)
	Color Doppler	Effective to be used in pregnancy identification at 17 days after mating	(129)
Infrared thermography		Detects temperature variations in the estrous cycle phases	(130)

*Source: research data*.

### Animal Breeding

For animal breeding, some papers identified genes linked to production characteristics. Others estimated genetic parameters or used computed tomography. The studies aimed primarily at supporting the selection of superior animals for breeding programs, and most of them involved the estimation of genetic parameters.

### Animal Behavior and Welfare

After applying the established criteria, there were no peer-reviewed scientific articles about Santa Inês sheep behavior. Only one study was selected about animal welfare, and it was related to stress at performing reproductive procedures (75).

### Animal Nutrition and Feeding

There was a wide range of studies involving the use of by-products as an alternative in the formulation of diets, mainly aiming at reducing costs. Other technologies deal with the supply of non-protein nitrogen sources, handling, and lipid supplementation. All these technologies are listed in Table 5.

**Table 5:** Technologies used to increase nutritional performance variables of Santa Inês sheep, 2017–2021.

**Technology**	**Description**	**Primary effect or outcome**	**Authors**
Dietary strategy	Cottonseed cake (CC)	Negative effect on nutritional performance variables (70, 140, and 210 g/kg of CC in DM)	(78)
	Coconut cake (COC)	Should be fed with caution (consumption of ether extract should not exceed 0.16% of body weight)	(79)
	Detoxified castor bean meal (DCBM)	Increased efficiency of feeding and rumination (21% of DCBM in DM)	(80)
	Licuri cake (LIC)	Improved production performance (174 g/kg of LIC in DM)	(81)
	Sunflower meal (SUM)	No positive effect on performance (10, 20, and 30% SUM in DM)	(82)
	Dehydrated distillers grains	Beneficial effect on performance variables (24% in DM)	(83)
	Babassu palm by-product –BPBP (*Attalea speciosa*)	Provides superior nutritional quality when enriched with broken maize and cassava scraping additives (48.75% of BPBP	(131)
	Guava (*P. guajava* L.) by-product	Maintains performance (16.4% in DM)	(84)
	Buriti oil	Improved performance variables (12 g/kg)	(85)
	Canola (CAO), sunflower (SUO) or castor oil (CO)	No productive increase (30 g of fatty acids-AG/kg DM of CAO, SUO or CO)	(86)
	Urea associated with cassava root, corn or spineless cactus	Did not improve performance	(88)
	Encapsulated nitrate	Can replace urea	(132)
Handling	Feeding frequency	Once daily feeding was recommended	(133)
	Volume and concentrate Ratio	The ratio 500:500 g/kg in hay-based diets was recommended for best performance	(134)
		A ratio of 600:400 g/kg in silage-based diets was recommended for best performance	(89)

*Source: research data*.

### Forage and Pasture

In this knowledge field, the studies addressed techniques of grazing systems and the use of different tropical or alternative forage plants as a technological increment for use in the production of Santa Inês sheep (Table 6).

**Table 6:** Technologies used in the forage and pasture field for Santa Inês sheep, 2017–2021.

**Technology**	**Description**	**Primary effect or outcome**	**Authors**
Grazing system	1-grazing sheep only, 2- grazing sheep after cattle, and 3-cattle and sheep grazing together	Simultaneous grazing (2) and isolated grazing (1) were recommended with the proposal of greater benefits	(90)
Tropical forage (pasture)	*Brachiaria brizantha*	The cultivars Marandu and Piatã were recommended for best production results	(94)
Cactus or alternative plant	Spineless cactus	Can replace Tifton 85 hay up to 50%	(135)
with forage potential	Spineless cactus	Can replace sugarcane up to 49.5%	(93)
Can replace up to 80%	Spineless cactus	Can replace wheat bran up to 80%	(95)
wheat bran	Spineless cactus associated with sugarcane	Can replace corn silage	(92)
Spineless cactus associated with sugarcane Can replace corn silag	Spineless cactus (SC) associated with maniçoba (*Manihot pseudogalziovii* Pax and Hoffman) hay	Alternative of use: 400 g/kg of SC and 300 g/kg of maniçoba hay (MH) or silage (MS).	(91)
Forage palm associated	Sisal (*Agave sisalana*, Perrine) Souza et al. (96)	Performance similar to Tifton hay	(96)
with maniocoba silage or hay Alternative of use: 400 g/kg of PF and 300 g/kg of maniocoba silage or hay	*Mimosa tenuiflora*	Can replace Buffel grass hay up to 20 g/100 g DM	(97)
Leguminous	*Leucaena leucocephala* e *Gliricidia sepium*	Can replace soybean meal	(98)
	*Macrotyloma axillare*	Potential use as a food source	(99)
Extruded forage		Can replace corn silage improving nutritional variables	(100)

*Source: research data*.

### Carcass and Meat Quality

The techniques and innovations aimed to improve, benefit, or not harm the quality of the carcass and meat of Santa Inês sheep. They were dietary strategies, supply of forage plants, and handling practices (Table 7).

**Table 7:** Technologies used in the carcass and meat quality evaluation for Santa Inês sheep, 2017–2021.

**Technology**	**Description**	**Primary effect or outcome**	**Authors**
Dietary strategy	Guava (*P. guajava* L.) by-product	Does not compromise the sensory characteristics of sheep meat (up to 30–40 % in DM)	(107, 108)
	Sunflower cake	Improved levels of nutraceutical parameters (30% in DM)	(101)
	Cottonseed (COS) combined with calcium lignosulfonate (CAL)	No effect on carcass characteristics (100 g/kg of COS and 100 g/kg of CAL)	(102)
	Cottonseed (COS) associated with chitosan	Increases unsaturated fatty acids in the meat (150 g/kg of COS and 136 mg chitosan/kg of body weight)	(103)
	Mazoferm	Can replace soybean meal without changing carcass characteristics (33, 67 and 100% in DM)	(104)
	Cassava wastewater (CWW)	Changes meat composition, but does not harm the overall quality (25, 50, 75 and 100% in DM or 0.5; 1 and 1.5 liters of CWW)	(105, 106)
	Canola grain	Does not influence meat composition and sensory attributes (8 and 16% in DM)	(109)
	Protein-energy supplementation	Favors carcass characteristics, but increases lipid oxidation (0.7% of BW)	(110)
	Protein-mineral supplementation	Improves carcass characteristics (0.7% of BW)	(120)
	Banana waste (BAW)	Does not interfere with meat characteristics and fatty acid profile (75% BAW)	(136)
	Babassu cake -BAC (*Orbignya speciosa*)	No effect on carcass characteristics and meat quality (up to 50% of BAC)	(137)
Cactus or alternative plant with forage potential	Spineless cactus (SC)	Improves lipid profile (30, 50, and 70% of SC or 17.6; 35.3; 53.2 and 71.1 % of SC)	(113, 114)
		No compromise on meat quality (33% of SC)	(115)
		Beneficial effect on most carcass traits (<75.5% of SC)	(116)
		Beneficial effect on most of the carcass traits (44% of SC)	(138)
	Spineless cactus combined with Tifton 85 hay or sugar cane bagasse	Improves sensory meat quality (35% of SC)	(117)
	*M. tenuiflora*	Improves meat characteristics (250 g/kg)	(118)
	Babassu leaves -BAL (*Orbignya phalerata*) and Mofumbo -MOF (*Combretum leprosum*)	Does not compromise carcass characteristics (33 g/100 g BAL and MOF)	(119)
Handling	Feed restriction (FRES)	Does not affect carcass yield (60% of FRES)	(121)
		Improves the lipid profile of the meat	(122)
		Improves fatty acid profile (30% of FRES)	(111)
	Castration	Promotes better lipid profile	(123)
	Castration and vitamin E supplementation (VES)	No significant effect (350 mg/kg of VES)	(124)
	Water salinity	No effect on carcass and meat characteristics (8,326 mgTDS/L)	(125)

*Source: research data*.

### Economics and Management of Livestock Systems

The lack of studies on economic analysis and feasibility of production systems of the Santa Inês breed was evidenced in this article. Therefore, it was possible to select only one study that performed this analysis within the production system of meat sheep raised on cultivated pastures (126).

## Discussion

The articles selected from those obtained from the databases after the refinement of the results represented 11.98%. This quantity reflects the efficiency of the search criteria used, which restricted the search by excluding most articles that were not within the focus of this systematic review. The Northeast and Southeast regions stood out with a higher frequency of work performed. These differ in technological level, since the first performs an activity still of subsistence and the second develops a production with a more advanced technical level, triggering better responses at the productivity level (9).

The prevalence of studies in the Northeast region may have been due to the breed selected in this study (Santa Inês), which is a hairless breed with great adaptation to warmer locations (12). On the other hand, the Southeast region has a milder climate and consumer demand for high-weight carcasses that provide bigger cuts and light fat cover (18). It can influence the implementation of experiments with more specialized and productive breeds for meat at the expense of adaptation, such as Dorper, White Dorper (19), Hampshire Down, Ile de France, Suffolk, and Texel, among others. In addition, they also end up using crossbreeds to benefit from the effect of heterosis (18, 20).

In recent years, most of the works with technological innovations are being developed in the Northeast that uses little or no technological increment (8–10). Therefore, the knowledge generated within research and education institutions needs to be carried to the countryside through a partnership among these institutions, technical assistance companies, and other government agencies. It implies not only disseminating but teaching the application of knowledge to sheep farmers.

The studies with potential technologies to be used within the production systems of meat sheep were especially those related to dietary strategies, pharmaceuticals, and handling tools. These techniques provide a productive increase by bringing benefits to health, reproduction, nutrition, and meat quality. The ones that gained prominence were the food resources, which came with the proposal of being easy to use through additives or low cost with by-products.

### Animal Health

Additives to sheep diets are associated with their biological power to provide health benefits to these animals. For example, red propolis has powerful biological activities and a high content of isoflavonoids bringing benefits to the defense cells and the energy status of sheep at the beginning of lactation (21). The probiotic *B. thuringiensis* did not interfere with animal performance, did not cause intoxication, and can be safely used with the daily addition of 2.5 × 106 spores/kg body weight (22). Another additive to sheep diets is chitosan (an alternative biopolymer to ionophores), which was recommended to be used with cottonseed husk since it would not affect blood metabolites, liver histopathological profile, and rumen papillae morphometry (23).

The safety of using cottonseed due to the presence of gossypol (an anti-nutritional factor) has been evidenced since this by-product did not cause health impacts to sheep when replacing soybean meal (24). Similarly, agro-industrial guava (*P.guajava* L.) waste promotes beneficial effects on the health of sheep when included up to 30% in the diet with a decrease in cholesterol concentration (25).

In general, the food additives and by-products presented here as technological tools contribute to improving animals' health, or their use does not cause harm to them. However, more studies need to be carried out with the Santa Inês species to prove their effectiveness.

The use of pharmaceuticals applied to animal health as a technology has been highlighted in two studies. The recombinant bovine somatotropin hormone used in ewes reduced the risk of pregnancy toxemia (26). However, gentamycin (an antibiotic used for intramammary drying therapy) does not prevent new infections nor cure previous subclinical mastitis (27). Therefore, only the use of the first drug would be recommended since the antibiotic in the second situation would only increase production costs.

Endoparasites have been a significant problem for sheep producers, as they reduce or limit production (28). Good animal nutrition may be able to generate resistance to prevent helminth infections. Such as protein supplementation that promoted early activation of the ovarian follicle in peripubertal ewes infected with *Haemonchus contortus* (29). The use of forages rich in condensed tannins has been recorded as a tool for helminths control in sheep, such as those present in *Acacia mearnsii* that could be considered an alternative to commercial anthelmintics (30). Other technologies used for helminth control are the FAMACHA© system which has significantly reduced the need for treatments in animals of various categories (31, 32). Biological control (nematophagous fungi) brought beneficial results by reducing infective gastrointestinal nematode larvae in pastures (28). The Famacha method is not a recent technique and has been used also in other species to identify susceptible (anemic) animals in the herd (33). On the other hand, biological control is a strategy that needs more research to have its efficiency proven.

### Animal Reproduction

The addition of substances for semen cryopreservation to maintain its viability and quality has been evidenced, bringing benefits to the freezing (34, 35) or cooling of semen (36). And as a dietary strategy, the supplementation with palm kernel cake does not affect seminal parameters and thus could be used as an alternative ingredient in sheep diets (37). As for the frozen/thawed sperm selection methods, swim-up would be the most advantageous if the goal is *in vitro* fertilization (38).

Nutrition can be a handling tool for body condition score recovery of ewes in different reproductive phases. In pre and postpartum, the supplementation of 1.5% of body weight with concentrate is recommended to support the maintenance of minerals in homeostasis and reproductive performance (39). In prepartum, supplementation of ADE vitamins improved energy metabolism (40). In the postpartum period, protected fat was not recommended because it reduced the probability of gestation (41). As for energy by-products, crude glycerin could be recommended for up to 10%, not affecting reproductive performance before and during the breeding season (42). In addition, it has a positive effect on the energy balance in the critical phases of late gestation and early lactation (43).

The use of hormones in the estrous synchronization of ewes through hormonal protocols is a technological tool to increase reproductive performance. Progesterone is the most commonly used in protocols but is also expensive. Thus, a study reporting the reuse of devices with this hormone evidenced that this practice would not affect pregnancy rates in synchronization protocols (44). Also aiming to reduce costs, Prates et al. (45) evaluated human intravaginal tampons soaked with human progesterone for induction and synchronization of estrus in Santa Inês ewes, verifying that these can be used alternatively for this purpose. Comparing the effect of short and long protocols based on progesterone and equine chorionic gonadotropin (eCG), Silva et al. (46) found that under the Amazon environment, the second one showed better results.

As well as progesterone, other ovulation inducers have been used in protocols, such as human chorionic gonadotropin (eCG) (47), gonadotropin-releasing hormone (GnRH) (48), and pFSH in combination with eCG (49) or successive administration (50).

Hormones have also been studied to aid in non-surgical transcervical embryo transfer. For example, a cervical dilation protocol consisting of oxytocin alone (51) and a hormone combination (estradiol esters, d-cloprostenol, and oxytocin) to induce cervical dilatation before non-surgical retrieval (52).

The combination of hormone dose and interval of use allows various protocols to be created. Therefore, each protocol is a different technology and the choice of one depends on the analysis of the production system.

To verify the effectiveness of using the *in vitro* embryo production technique, Ferreira-Silva et al. (53) compared it with natural mating but observed no difference between the two methods studied.

Some management tools can also increment reproductive efficiency. For example, the exposure of the male to the female with temporary weaning for 24 h improved the reproductive performance in ewes under postpartum anestrous (54). Also, the male effect promotes pre-ovulatory LH peaks up to 80 h after physical contact (55).

Seeking to facilitate calf handling, dexamethasone in ewes at 145 days gestation (8 and 16 mg) is an effective alternative to induce labor and concentrate handling and attention to the neonates without altering labor (127).

Imaging and temperature measuring devices can assist reproductive handling as not invasive tools. They also can provide an immediate result, helping the agricultural science professional in decision-making. The study by Beltrame et al. (128) found that the manual evaluation allows more accurate identification of the onset and end of systole and diastole. Color Dopplers are also a reliable tool starting 17 days after mating to distinguish between pregnant and non-pregnant animals (129). Finally, infrared thermography has been employed in sheep reproduction and has proven efficient for detecting small temperature variations during different phases of the estrous cycle (130).

### Animal Breeding

#### Genes Related to Production Traits

The identification of genes associated with quantitative production traits aims at superior animals' selection for certain desirable traits. Thus, variants of μ-calpain and calpastatin genes responsible for protein renewal are associated with the body (56) and carcass traits in Santa Inês sheep. These are new sources of information to improve these traits through selection schemes (57). Variants of the Myostatin (MSTN) and MyoD family genes, which play vital roles in myogenesis, have been found. These, in turn, may be associated with the physicochemical characteristics of meat in Santa Inês sheep (58).

In the field of reproduction, a new allele of Growth and Differentiation Factor 9, called FecG(E) (Embrapa), has been found, and ewes have to have a higher frequency of morphologically normal multiple oocytes (59).

#### Estimation of Genetic Parameters

Genetic and phenotypic changes are relevant for animal breeding programs and can be achieved through information from estimated genetic parameters (60). Several authors have estimated the heritability of economic interest traits in the production system of Santa Inês sheep. Low heritabilities (<0.1) result in small gains in the selection. On the other hand, medium (>0.1 and <0.3) to high (>0.3) heritabilities indicate that genetic progress can be achieved by selection based on the traits studied. Then, heritability as well as other genetic parameters such as correlation and repeatability provide support for genetic selection (61).

Examples of low heritability have been found for the traits of plasma urea concentration, used as an indicator of nitrogen utilization and excretion (62), and the number of eggs per gram of feces (a worm-resistance trait) (63, 64). Nevertheless, medium to high heritability was identified in carcass characteristics, fatty acid profile (60, 65), growth (66, 67), worm resistance, and FAMACHA grade (63, 67).

Through correlation, it was possible to predict the physicochemical compositions of carcasses from measurements *in vivo* and on the carcass (68). Biometric measurements were also able to accurately predict hot carcass weight, cold carcass weight, and the weight of primary carcass cuts (69).

Repeatability refers to the expression of the same trait at different times in the life of the same animal. In seminal characteristics of sheep, it was identified as low repeatability. Thus, multiple evaluations of semen characteristics are recommended for selection (70). However, repeatability estimation for the FAMACHA score was medium. It shows a high correlation with other worm resistance traits, indicating its potential to be included as a criterion for sheep genetic selection for endoparasite resistance (63).

The inclusion of genomic information has shown the potential to increase the accuracy of prediction of genetic values and speed selection. The inclusion of this information has increased the estimated heritability of carcass characteristics (71) and traits that indicate resistance to gastrointestinal nematode infection (72, 73).

#### Computerized Tomography

Computerized tomography has been identified as an essential tool in selecting animals with the best reproductive characteristics. It allows an evaluation of tissues from live animals and detects changes during the growth phase. In addition, it reveals differences in tissue composition between males and females (74).

### Animal Behavior and Welfare

Research in animal behavior and welfare has been infrequently approached. Studies in this field are valuable because animals are adaptable to the environment and, in the presence of stressful agents, this may directly affect performance, impacting the entire production system (76). In this way, technologies capable of extinguishing or mitigating these variables that compromise well-being become of utmost importance.

This research needs to take into consideration the concept of the “five freedoms” to guarantee a minimum quality of life for animals: (1) Physiological freedom (freedom from hunger, thirst, and malnutrition); (2) Environmental freedom (physical environment suitable to the species); (3) Sanitary freedom (freedom from disease, wounds, and pain); (4) Behavioral freedom (the possibility to manifest behavioral characteristics proper to the species), and; (5) Psychological freedom (freedom from fear and discomfort) (77).

A single study for this review was selected, which used tools related to reproductive biotechniques. It showed that transcervical and laparotomy techniques affected the welfare of the ewes in embryo collection, but the first one was less stressful (75).

### Animal Nutrition and Feeding

By-products with protein nutritional value have widely experimented on the studies. However, supplementation with cottonseed cake and coconut meal needs to be used with caution as it may decrease animal performance variables (78, 79). Castor meal and licuri cake are also by-products with the potential to be used as a protein source (80, 81). On the other hand, sunflower meals did not show satisfactory results regarding performance and other characteristics as a protein alternative (82).

Aiming to replace soybean meal with dehydrated distillers grains in the performance and carcass quality of confined lambs, Gomes et al. (83) recommend its use in up to 24% replacement in the dry matter of the diet. Another protein feed to provide to sheep is silage based on by-products from the industrialization of babassu palm (*A. speciosa*) with the inclusion of additives such as corn chips and cassava shavings, recommended by Codognoto et al. (131).

Regarding by-products with energy values, the replacement of corn with guava by-products was indicated to be included in the diet of sheep in confinement (84). Also, fats and oils have been used in ruminant feed to increase the energy density of the diet. Then, the supplementation with buriti oil improves consumption, digestibility, and animal performance (85). However, the use of other oils, such as canola, sunflower, or castor oil, did not provide milk yield or increase the final bodyweight of the lambs (86).

In this way, by-products become an alternative capable of reducing production costs with animal feed without compromising their performance in general. However, an inclusion limit must be respected as these foods may have antinutritional factors.

For maximizing microbial protein synthesis in the rumen, urea (source of nitrogen for the production of amino acids) needs to be associated with a rapidly degrading carbohydrate source to supply energy to the microorganisms. This association aims to replace all or part of a true protein source, such as soybean meal (87). However, the association of urea with cassava root, corn, or forage palm could not replace soybean meal in the diet of sheep in confinement (88). Also, encapsulated nitrate did not influence intake or nutrient digestibility replacing urea in lamb diets (132).

Feeding management is also able to influence animal performance. By evaluating the effects of feeding frequencies (once, twice, three times, and four times a day), Saldanha et al. (133) could recommend feeding once a day to avoid compromising the performance of confined male lambs.

The ratio of roughage and concentrate (V:C) can also be considered a handling strategy to improve performance and prevent metabolic problems. In hay-based diets, the ratio of 500:500 g/kg improved both performance and feed efficiency in lambs (134). For diets formulated with silage, it is recommended 600:400 g/kg for the highest weight gain (89).

### Forage and Pasture

Three different grazing systems (1-sheep grazing only, 2-sheep grazing after cattle, and 3-cattle and cattle grazing together) were studied by Santos et al. (90), who found that simultaneous-3 and isolated-1 resulted in the best forage production and animal performance.

Tropical forages are grass species that have the advantage of being adapted to the most adverse conditions. However, they are generally less productive. In a grazing system, Marandu and Piatã cultivars (*Brachiaria brizantha)* allowed better productive performance and body development of Santa Inês sheep (94). The spineless cactus occupies a prominent position in the diet composition of ruminants and is the most widely cultivated cactus in the Brazilian semi-arid region. However, the exclusive supply of this plant is not recommended, due to its low dry matter content and high degradability (134), but its use has been an alternative feed on sheep diet (91–93, 95, 135). Sisal (*Agave* sp) also is a cactus that can be present in sheep feed through its by-product, and its pulp provided lamb performance similar to those with Tifton hay-based diets, also reducing costs (96).

Still using plants adapted to semi-arid conditions, there have been reports of the *M.tenuiflora* (Willd.) hay (97). Also, the Leucaena (*Leucaena leucocephala*.), Gliricidia (*Gliricidia sepium*), and Macrotiloma (*Macrotyloma axillare*) leguminous plants were used as a source of protein (98, 99).

According to the works presented, it is evident that tropical forages and semi-arid cactus are viable alternatives for feeding Santa Inês sheep as they have good adaptation and are available in periods of greater scarcity. However, attention should be paid to the fact that these forages are less productive and, in the case of cactus, with high humidity. Therefore, it could compromise the consumption and performance of the animals. Another technology employed in the feed management of meat sheep can be demonstrated by Oliveira et al. (100) when they verified that bulking extrusion (heat treatments that modify physical and chemical properties) improved nutritional parameters when replacing corn silage in the diet of sheep.

### Carcass and Meat Quality

Evaluations of carcass and meat quality from the supplementation with by-products were the majority of the selected studies in Santa Inês sheep. These studies aim to identify whether the use of these foods in the diet would cause any modification in carcass and meat characteristics.

As a protein feed, sunflower cake and cottonseed, combined with calcium lignosulfonate (a cellulose industry by-product) or with an antimicrobial (chitosan), can be included in sheep diets without overall harm to the carcass and meat quality (101–103). Another alternative protein feed would be mazoferm (a residue from the processing of corn) that can replace soybean meal without altering the carcass characteristics (104).

Some studies have addressed cassava wastewater (manipueira) as a source of energy in sheep diets. However, its use, among other effects, alters the physicochemical composition and fatty acid profile of the meat, not compromising the overall meat quality (105, 106). On the other hand, the supplementation with guava (*P.guajava* L.) agro-industrial by-products does not compromise the sensory characteristics of sheep meat if added in up to 30 to 40% (107, 108). And still considering energy by-products, despite being rich in unsaturated fatty acids, canola grain did not influence instrumental analysis, centesimal composition, and sensory attributes of meat (109).

The effect of protein-energy supplementation on carcass characteristics of sheep has also been verified. Nevertheless, despite favoring meat characteristics, this type of supplementation may increase lipid oxidation during the aging time and affect consumer acceptance (110). However, protein-mineral supplementation could improve the quantitative and qualitative carcass characteristics (120).

Some authors have studied feed restriction as a strategy that could benefit carcass quality and reduce system costs. Feed restriction of up to 60% does not affect carcass yield in sheep (121), improving meat quality and lipid profile (122). However, Campos et al. (111) recommend a lower dietary restriction (30%).

In an attempt to find an additional feed source, Luz et al. (137) found that babassu has the potential to replace elephant grass up to 50% without causing effects on carcass characteristics or meat quality. It was possible because of the by-product characteristics of bulky feed with 62.30% neutral detergent fiber, 19.06% crude protein, and 9.20% ethereal extract (112). Also, the supply of banana leftovers can cheapen the diet in places with availability, and its inclusion can replace up to 75% of corn bran (136).

The effects of supplementing sheep with spineless cactus, as well as in performance evaluation, were observed under meat quality. For instance, it improved lipid profile (113, 114) and meat sensory quality (117). In addition, this cactus could replace maniçoba (*M. pseudogalziovii* Pax and Hoffman) hay by up to 33% with increased carcass finishing fat without compromising meat quality (115). Another recommendation was to include <75.3% palm in sheep diets, replacing Tifton hay (116) or 44% palm, replacing sugarcane (138), with the proposal to obtain beneficial effects on most carcass traits.

Another plant adapted to semi-arid conditions is the legume *M. tenuiflora*. It can replace *Brachiaria decumbens* cv. Basilisk hay with the proposal of improving several meat characteristics (118). Also, when using diets based on plants from the Brazilian caatinga, Abdalla Filho et al. (119) observed that feeding babassu and mofumbo did not compromise the carcass characteristics and the meat fatty acid profile.

When verifying the effect of vitamin E supplementation and castration in Santa Inês lambs, Torres-Geraldo et al. (124) found no significant changes in meat quality characteristics. However, the technologies applied are not justified. On the other hand, Lima et al. (123) found that non-castrated animals produce better quality meat for human health, with a better lipid profile.

Regarding water management, when studying salinity levels, it was found that salinity levels up to 8,326 mg TDS/L do not affect the carcass and meat characteristics of Santa Inês lambs (125).

Therefore, most of the techniques presented to contribute or not alter carcass and meat quality involve animal feed or feed restriction. Nonetheless, the level of inclusion or restriction must be respected so that they do not bring negative results.

### Economics and Management of Livestock Systems

We highlight the need for economic approaches in studies involving the production systems of Santa Inês sheep. Economic analysis is essential because it identifies the items that should be part of the activity costs and the factors that compromise the system's profitability. It also describes the importance of productive indexes evaluation to identify precisely where the system is being harmed and contributes to generating solutions for greater efficiency and activity profitability (139).

The study identified in this review performed the economic analysis of sheep production systems in pastures of different cultivars of *Panicum maximum* (Aruana and Massai) and *Brachiaria brizantha* (Marandu and Piatã). The authors calculated mainly revenue, costs, annual operating profit, and internal rate of return, verifying greater feasibility and attractive remuneration rates in production systems of meat sheep on marandu and massai pastures (126).

## Conclusion

Most technologies were in northeastern states. However, since this region has a subsistence production system, it is inferred that farmers have not been using the studied tools. Thus, government agencies should develop public policies to disseminate the techniques in rural areas. Also, it needs an engagement of universities in diffusing the knowledge generated therein to sheep producers.

It was also possible to identify the scarcity of studies that present technologies in animal welfare and behavior fields. On the other hand, several papers used a by-product as a dietary strategy to evaluate the most different knowledge fields (health, reproduction, nutrition, meat quality) and used the cost reduction argument. Nevertheless, they did not perform any economic analysis to support this justification. Furthermore, studies focusing on the economic evaluation of production systems were scarce. Thus, research in this knowledge field is needed, especially in profitability system evaluation.

## Data Availability Statement

The original contributions presented in the study are included in the article/supplementary material, further inquiries can be directed to the corresponding author/s.

## Author Contributions

ASB conceived, designed the study, searched the literature, extracted the data, and wrote the manuscript. ASB, MASS, and JBL-J analyzed and interpreted the data. MASS and JBL-J critically revised the manuscript. All authors have read and approved the final manuscript.

## Funding

This study received financial support for publication fee from Pró-Reitoria de Pesquisa e Pós-Graduação (PROPESP/UFPA). Also, ASB received scholarship from the Coordenação de Aperfeiçoamento de Pessoal de Nível Superior (CAPES) – Brasil (Finance Code 001).

## Conflict of Interest

The authors declare that the research was conducted in the absence of any commercial or financial relationships that could be construed as a potential conflict of interest.

## Publisher's Note

All claims expressed in this article are solely those of the authors and do not necessarily represent those of their affiliated organizations, or those of the publisher, the editors and the reviewers. Any product that may be evaluated in this article, or claim that may be made by its manufacturer, is not guaranteed or endorsed by the publisher.
